# Identification of (*Z*)-2,3-Diphenylacrylonitrile as Anti-Cancer Molecule in Persian Gulf Sea Cucumber *Holothuria parva*

**DOI:** 10.3390/md15100314

**Published:** 2017-10-16

**Authors:** Salimeh Amidi, Zahra Hashemi, Abbasali Motallebi, Melika Nazemi, Hoda Farrokhpayam, Enayatollah Seydi, Jalal Pourahmad

**Affiliations:** 1 Faculty of Pharmacy, Shahid Beheshti University of Medical Sciences, 19839-63113 Tehran, Iran; s.amidi@sbmu.ac.ir (S.A.); zahrahashemi964@rocketmail.com (Z.H.); Hodaa.farrokhpayam@gmail.com (H.F.); 2 Ministry of Jihad-e-Agriculture, Research and Education and Extension Organization (AREEO) and The Iranian Fisheries Research Organization, 14155-6116 Tehran, Iran; motalebi@ifro.ir; 3 Persian Gulf and Oman Sea Ecological Center, Iranian Fisheries Science Research Institute, Agricultural Research, Education and Extension Organization (AREEO), Bandar Abbas, Iran; melikanazemi@yahoo.com; 4 Research Center for Health, Safety and Environment (RCHSE), Alborz University of Medical Sciences, Karaj, Iran; 5 Department of Occupational Health Engineering, Alborz University of Medical Sciences, Karaj, Iran

**Keywords:** hepatocellular carcinoma, *Holothuria parva*, mitochondria, mass analysis, (*Z*)-2,3-diphenylacrylonitrile

## Abstract

Hepatocellular carcinoma (HCC), also named cancerous hepatoma, is the most common type of malignant neoplasia of the liver. In this research, we screened the Persian Gulf sea cucumber *Holothuria parva* (*H. parva*) methanolic sub-fractions for the possible existence of selective toxicity on liver mitochondria isolated from an animal model of HCC. Next, we purified the most active fraction. Thus the structure of the active molecule was identified. HCC was induced by diethylnitrosamine (DEN) and 2-acetylaminofluorene (2-AAF) protocol. Rat liver mitochondria for evaluation of the selective cytotoxic effects of sub-fractions of *H. parva* were isolated and then mitochondrial parameters were determined. Our results showed that C1 sub-fraction of methanolic extract of *H. parva* considerably increased reactive oxygen species (ROS) generation, collapse of mitochondrial membrane potential (MMP), swelling in mitochondria and cytochrome c release only on HCC liver mitochondria. Furthermore, the methanolic extract of *H. parva* was investigated furthermore and the active fraction was extracted. In this fraction, (*Z*)-2,3-diphenylacrylonitrile molecule, which is also known as α-cyanostilbene, was identified by mass analysis. This molecule increased ROS generation, collapse of MMP, swelling in mitochondria and finally cytochrome c release only on HCC liver mitochondria. The derivatives of (*Z*)-2,3-diphenylacrylonitrile in other natural products were also reported as an anti-cancer agent. These results suggest the eligibility of the (*Z*)-2,3-diphenylacrylonitrile as a complementary therapeutic agent for patients with HCC.

## 1. Introduction

Cancer is one of the major causes of mortality in the world [1]. Hepatocellular carcinoma (HCC), an early cancer of hepatocytes, is considered the fifth most common fatal malignant tumor that is not detected at the early phase in the world [1,2,3]. Environmental toxic chemicals, air and water pollutants, food additives, hepatitis B or hepatitis C, obesity and non-alcoholic fatty liver diseases are identified risk factors of HCC [2,4]. The approved treatments for detected HCC are chemotherapy and liver transplantation, in which patients have poor tolerance due to adverse effects [4]. Thus, it is necessary to find an effective anti-tumor agent with fewer side effects.

It is known that natural products and their derivatives were the source of 79.8% of all approved anti-cancer drugs between 1981 and 2010 [5]. In searching for novel anti-cancer agents among natural products, marine organisms can be considered as a source of versatile biological compounds that could target many cellular pathways [6]. It was reported that, from 1940 to 2010, three anti-cancer drugs with marine origin were introduced to the market:e Starsaid^®^ (cytarabine, introduced in 1993), Yondelis^®^ (trabectedin, 2007) and Halaven^®^ (eribulin, 2010). Currently, there are many marine anti-cancer molecules in the pre-clinical and clinical trial phases [5]. These data demonstrate the promising role of marine products in anti-cancer drug development.

The sea cucumber is a marine animal belonging to the Echinoderm phylum. This animal contains several bioactive molecules such as alkaloids, steroids, terpens and peptides [7]. Anti-tumorigenic properties of triterpene glycosides isolated from sea cucumbers (*Mensamaria intercedens*) have been previously evaluated [8]. In addition, the anti-tumor and anti-metastatic effects of two sulfated triterpene glycosides were isolated from sea cucumber species (*Pearsonothuria graeffei*) have been reported [8]. In addition, a triterpenoid glycoside (Frondoside A) was isolated from sea cucumber and its anti-proliferative effects on human pancreatic cancer cell lines, AsPC-1 and S2013, were observed [8]. Organic extract of *Holothuria scabra* demonstrated anti proliferative effect against human A549 non-small lung cancer cells and C33A cervical cancer cells [8]. We previously reported the toxicity of *Holothuria parva* methanolic extract against mitochondria of HCC hepatocytes, and showed that mitochondrial targeting is the important mechanism by which *H. parva* can potentially and selectively induce apoptosis in HCC hepatocytes. In this research, we aimed to figure out the bioactive molecule in *H. parva* methanolic sub-fractions, which may be promising treatment for HCC.

In this study, we screened the selective toxicity of Persian Gulf sea cucumber (*H. parva*) methanolic sub-fractions on liver mitochondria isolated from animal model of HCC. The most active fraction is then purified and the structure of active molecule was identified.

## 2. Results

### 2.1. Fractionation

**Figure 1:**
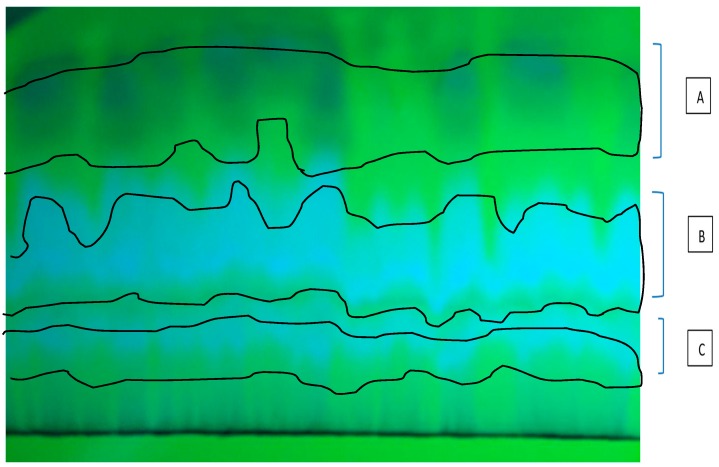
Thin layer chromatography (TLC) of fractionation methanolic extract using MeOH:CHCl_3_ (7:3) as mobile phase. The first ultraviolet (UV)-distinct band from top was named A, the second B, and the third C.

As the results of the first thin layer chromatography (TLC) related to methanolic extract using MeOH:CHCl_3_ (7:3) as mobile phase, three ultraviolet (UV)-distinct bands from top to bottom on the plate, in terms of polarity, were observed (Figure 1). Each of these bands from the highest polarity to the lowest demonstrated one fraction: A, B and C. The determination of mitochondrial parameters such as increase in the reactive oxygen species(ROS) formation and mitochondrial swelling were done with the 250, 500 and 1000 μg/mL concentrations of these fractions. The results showed no evidence of apoptosis by mitochondrial pathway by the fractions A and B in the HCC group, thus we continued to work with C.

**Figure 2:**
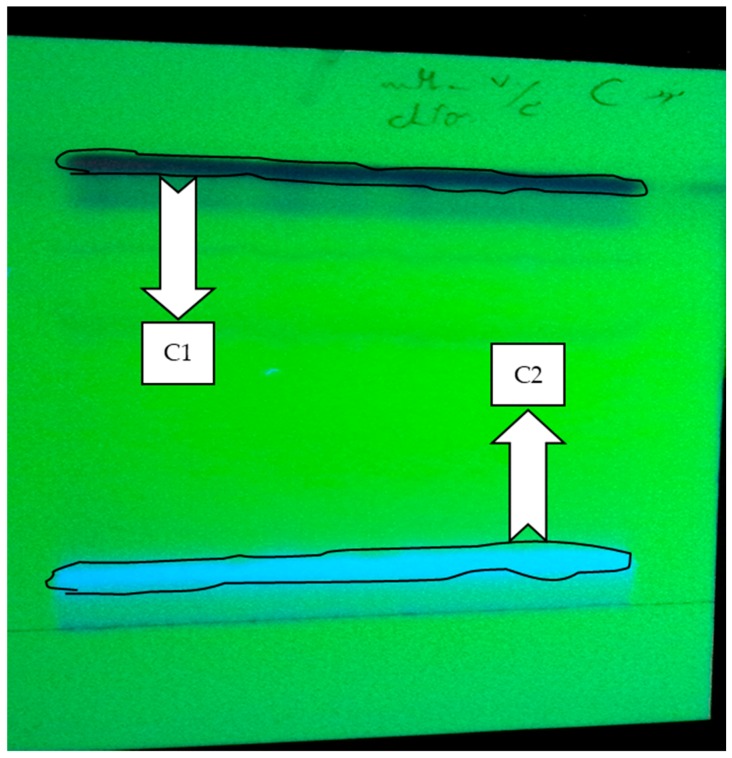
The fractionation of the active fraction (C) using MeOH:CHCl_3_ (7:3) as mobile phase by TLC plate method. C1 was the first UV-distinct band, and C2 was the second UV-distinct band.

The active fraction was re-chromatographed on TLC plate with MeOH:CHCl_3_ (7:3) and C1 and C2 were the obtained sub-fractions by polarity on the plate (Figure 2). After re-testing of mitochondrial parameters, C2 had no considerable results in ROS formation, mitochondrial swelling, determination of MMP and cytochrome c release in the HCC group, while C1 showed desirable results, thus this sub-fraction was accepted as an effective section of the methanolic *H. parva* extract.

In searching for one effective anti-cancer molecule in this extract, the identification process continued with sub-fraction C1.

### 2.2. H. parva (C1 Sub-Fraction) Increased Mitochondrial ROS Level

**Figure 3:**
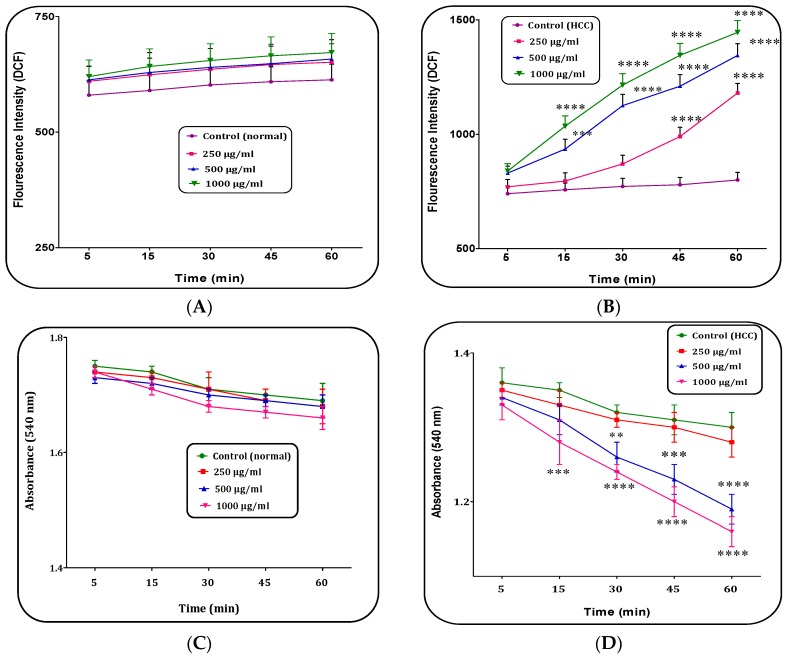
The mitochondrial reactive oxygen species (ROS) level assay. The effect of C1 sub-fraction of methanolic extract of *H. parva* (250, 500 and 1000 μg/mL) on mitochondrial ROS generation in: the normal mitochondria (**A**); and the HCC mitochondria (**B**) obtained from liver hepatocytes within 60 min of incubation. Mitochondrial Swelling assay. The effect of C1 sub-fraction of methanolic extract of *H. parva* (250, 500 and 1000 μg/mL) on mitochondrial swelling in: the normal mitochondria (**C**); and the HCC mitochondria (**D**) obtained from liver hepatocytes within 60 min of incubation. Data are shown as mean ± SD (*n* = 3). (**A**,**C**) Control means vehicle treated normal mitochondria. (**B**,**D**) Control means vehicle treated HCC mitochondria. **, *** and **** show significant differences in comparison with corresponding control (HCC) groups for *p* < 0.01, *p* < 0.001 and *p* < 0.0001, respectively.

The fractions A, B and C2 of *H. parva* (250, 500 and 1000 μg/mL) did not significantly change ROS generation on the liver mitochondria isolated from HCC induced rats within 60 min of incubation (data not shown). On the other hand, different concentrations (250, 500 and 1000 μg/mL) of C1 sub-fraction of the extract significantly (*p* < 0.05) increased ROS generation only in the mitochondria obtained from the HCC, but not normal mitochondria (Figure 3A,B).

### 2.3. H. parva (C1 Sub-Fraction) Induced Mitochondrial Swelling

Our results showed that fractions A, B and C2 of *H. parva* (250, 500 and 1000 μg/mL) methanolic extract did not significantly increase mitochondrial swelling on HCC liver mitochondria within 60 min of incubation (data not showing). Mitochondrial swelling is the well-known indicator of mitochondrial MPT pore opening, the starting event in mitochondria mediated apoptosis. Fraction C showed significant results. As shown in Figure 3D, the additional C1 sub-fraction (active fraction) of methanolic extract at concentrations of 500 and 1000 μg/mL induced swelling in mitochondria obtained from the HCC group. Nevertheless, addition of same fraction of the extract at the above mentioned concentrations did not significantly change mitochondrial swelling in the normal liver mitochondria (Figure 3C).

### 2.4. H. parva Extract (C1 Sub-Fraction) Induced Collapse on the Mitochondrial Membrane Potential (MMP)

**Figure 4:**
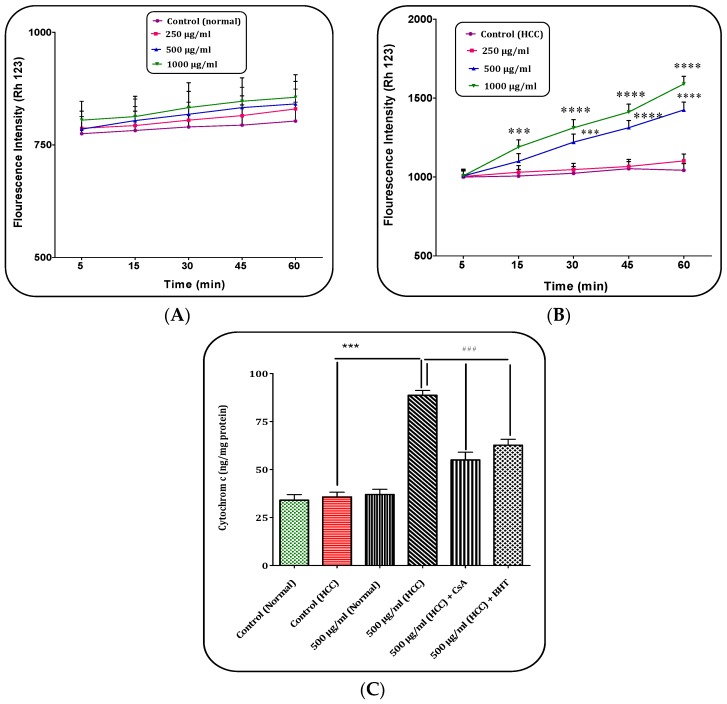
The Mitochondrial Membrane Potential (MMP) assay. The effect of C1 sub-fraction of methanolic extract of *H. parva* (250, 500 and 1000 μg/mL) on MMP collapse in: the normal mitochondria (**A**); and the HCC mitochondria (**B**) obtained from liver hepatocytes at within 60 min of incubation. The cytochrome c release assay. (**A**) Control means vehicle treated normal mitochondria. (**B**) Control means vehicle treated HCC mitochondria. *** and **** indicate significant differences in comparison with corresponding control (HCC) groups for *p* < 0.001 and *p* < 0.0001, respectively. (**C**) The effect of C1 sub-fraction of methanolic extract of *H. parva* (500 μg/mL) on cytochrome c release in the normal mitochondria and the HCC mitochondria obtained from liver hepatocytes at within 60 min of incubation. Data are shown as mean ± SD (*n* = 3). *** indicate significant differences in comparison with corresponding control (HCC) groups for *p* < 0.001. ^###^ Significant differences in the comparison with 500 μM (HCC) C1 sub-fraction (*p* < 0.001).

Our results showed that (Figure 4B), C1 sub-fraction of the extract at the highest concentrations (500 and 1000 μg/mL) selectively induced collapse of MMP only in the mitochondria obtained from the HCC hepatocytes. Furthermore, at low concentrations (250 μg/mL), C1 sub-fraction did not induce collapse of MMP within 60 min of incubation (Figure 4A).

### 2.5. H. parva Extract (C1 Sub-Fraction) Increased Cytochrome c Release

The obtained results indicated that C1 sub-fraction of *H. prava* extract significantly (*p* < 0.05) decreased mitochondrial swelling and MMP and it is expected that C1 sub-fraction could induce the release of cytochrome c from the mitochondria. As shown in Figure 4C, C1 sub-fraction of extract (500 μg/mL) significantly (*p* < 0.05) induced release of cytochrome c (ng/mg mitochondrial protein) only in the mitochondria isolated from HCC hepatocytes group. In addition, pre-treatment of mitochondria with cyclosporine A (CsA), as MPT inhibitor, and butylated hydroxyl toluene (BHT), as an antioxidant, significantly inhibited cytochrome c release (*p* < 0.05) from mitochondria when encountered to C1 sub-fraction of *H. prava* (500 μg/mL). These results showed the direct role of oxidative stress and MPT pore opening in cytochrome c release resulting from exposure to C1 sub-fraction of *H. parva* methanolic extract.

### 2.6. Determination of the Component in C1 Sub-Fraction of H. parva Methanolic Extract

**Figure 5:**
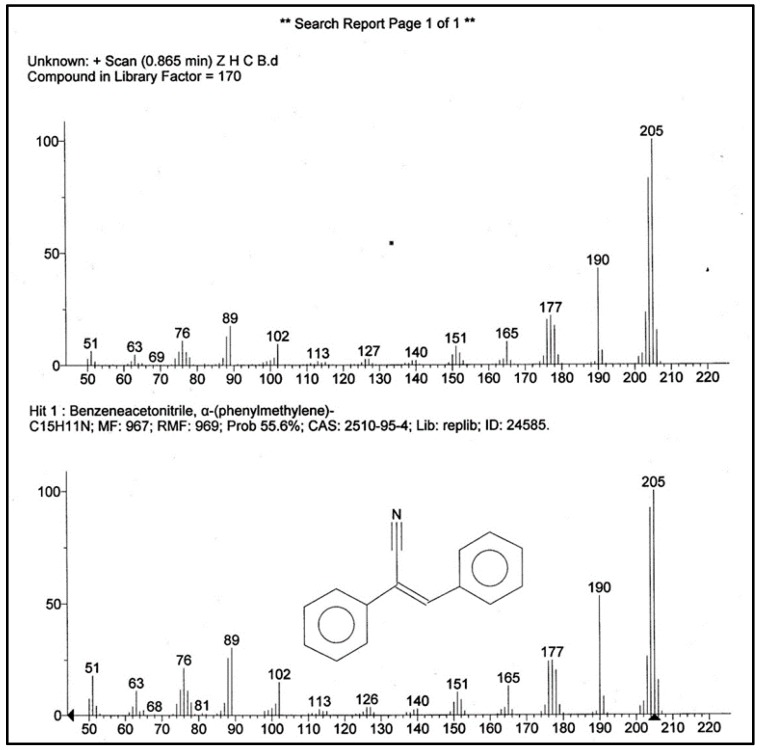
The mass (MS) Analysis of the C1 Sub-fraction.

C1 sub-fraction showed a molecular ion at *m*/*z* 205 in mass spectrum. The obtained spectrum for C1 was searched and compared with the spectrum of known components stored in the National Institute Standard and Technology (NIST) library (version 2.0f and NIST/EPA/NIH Mass Spectral Library 2008). The result showed a match for (*Z*)-2,3-diphenylacrylonitrile (Figure 5).

### 2.7. (Z)-2,3-Diphenylacrylonitrile Increased Mitochondrial ROS Level

**Figure 6:**
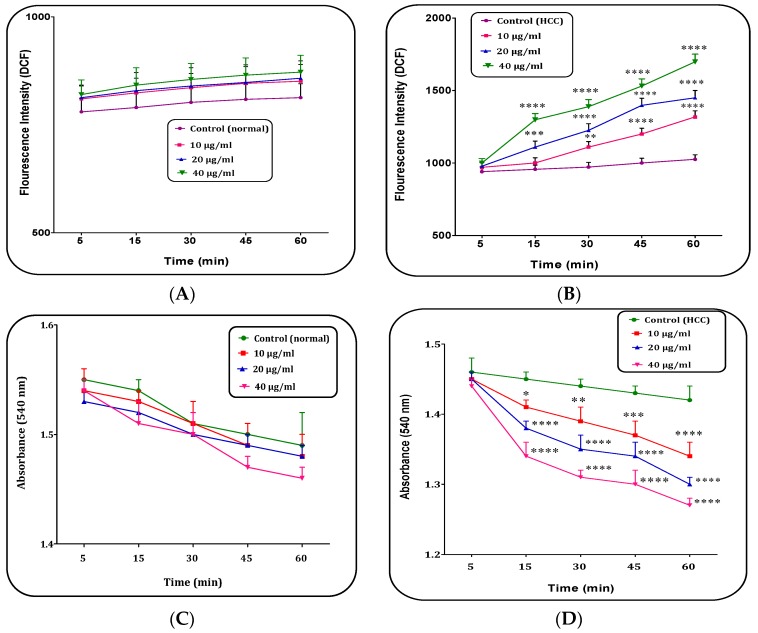
The mitochondrial reactive oxygen species (ROS) level assay. The effect of (*Z*)-2,3-diphenylacrylonitrile of *H. parva* (10, 20 and 40 μg/mL) on mitochondrial ROS generation in: the normal mitochondria (**A**); and the HCC mitochondria (**B**) obtained from liver hepatocytes within 60 min of incubation. Mitochondrial Swelling assay. The effect of (*Z*)-2,3-diphenylacrylonitrile of *H. parva* (10, 20 and 40 μg/mL) on mitochondrial swelling in: the normal mitochondria (**C**); and the HCC mitochondria (**D**) obtained from liver hepatocytes within 60 min of incubation. Data are shown as mean ± SD (*n* = 3). (A,C) Control means vehicle treated normal mitochondria. (B,D) Control means vehicle treated HCC mitochondria. *, **, *** and **** show significant differences in comparison with corresponding control groups for *p* < 0.05, *p* < 0.01, *p* < 0.001 and *p* < 0.0001, respectively.

Our results showed that several concentrations (10, 20 and 40 μg/mL) of (*Z*)-2,3-diphenylacrylonitrile from *H. parva* extract significantly (*p* < 0.05) increased ROS generation only in the mitochondria isolated from the HCC group (Figure 6A,B).

### 2.8. (Z)-2,3-Diphenylacrylonitrile Induced Mitochondrial Swelling

As shown in Figure 6D, the addition of all (*Z*)-2,3-diphenylacrylonitrile applied concentrations induced swelling in mitochondria isolated from the HCC group. However, this compound at all concentrations did not significantly induce mitochondrial swelling in the normal mitochondria (Figure 6C).

### 2.9. (Z)-2,3-Diphenylacrylonitrile Induced Collapse on the Mitochondrial Membrane Potential (MMP)

**Figure 7:**
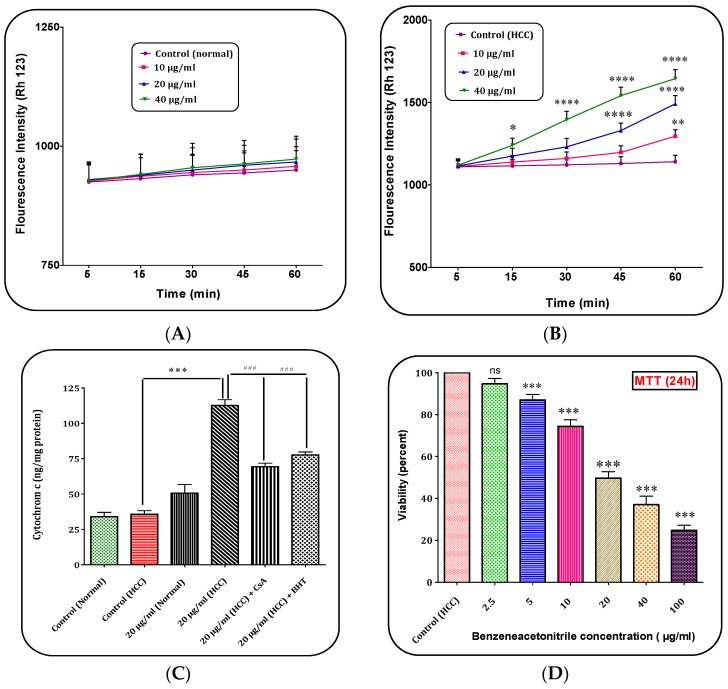
The Mitochondrial Membrane Potential (MMP) assay. The effect of (*Z*)-2,3-diphenylacrylonitrile of *H. parva* (10, 20 and 40 μg/mL) on MMP collapse in: the normal mitochondria (**A**); and the HCC mitochondria (**B**) obtained from liver hepatocytes at within 60 min of incubation. (**A**) Control means vehicle treated normal mitochondria; (**B**) Control means vehicle treated HCC mitochondria; (**C**) The cytochrome c release assay. The effect of (*Z*)-2,3-diphenylacrylonitrile of *H. parva* (20 μg/mL) on cytochrome c release in the normal mitochondria and the HCC mitochondria obtained from liver hepatocytes at within 60 min of incubation; (**D**) MTT assay: Hepatocytes obtained from HCC group were treated with (*Z*)-2,3-diphenylacrylonitrile of *H. parva* (0, 2.5, 5, 10, 20, 40 and 100 μg/mL) and incubated for 24 h. Data are shown as mean ± SD (*n* = 3). *, **, *** and **** indicate significant differences in comparison with corresponding control (HCC) groups for *p* < 0.001 and *p* < 0.0001, respectively. ^###^ Significant differences in the comparison with 20 μg/mL (HCC) (*Z*)-2,3-diphenylacrylonitrile (*p* < 0.001).

Our results showed that (*Z*)-2,3-diphenylacrylonitrile at concentrations of 10, 20 and 40 μg/mL induced collapse of MMP only in the mitochondria obtained from the HCC hepatocytes (Figure 7A,B).

### 2.10. (Z)-2,3-Diphenylacrylonitrile Increased Cytochrome c Release 

As shown in Figure 7C, (*Z*)-2,3-diphenylacrylonitrile at concentrations of 20 μg/mL significantly induced release of cytochrome *c* selectively in the mitochondria isolated from HCC hepatocytes group. Furthermore, pre-treatment of mitochondria with CsA and BHT significantly inhibited cytochrome c release from mitochondria when treated with (*Z*)-2,3-diphenylacrylonitrile (20 μg/mL).

### 2.11. (Z)-2,3-Diphenylacrylonitrile Decreased Cell Viability

Our results in Figure 7D showed that (*Z*)-2,3-diphenylacrylonitrile at concentration of 5, 10, 20, 40 and 100 μg/mL significantly decreased cell viability in hepatocytes obtained from HCC rats.

### 2.12. Mix Probe between Synthesized (Z)-2,3-Diphenylacrylonitrile and C1 Fraction 

**Figure 8:**
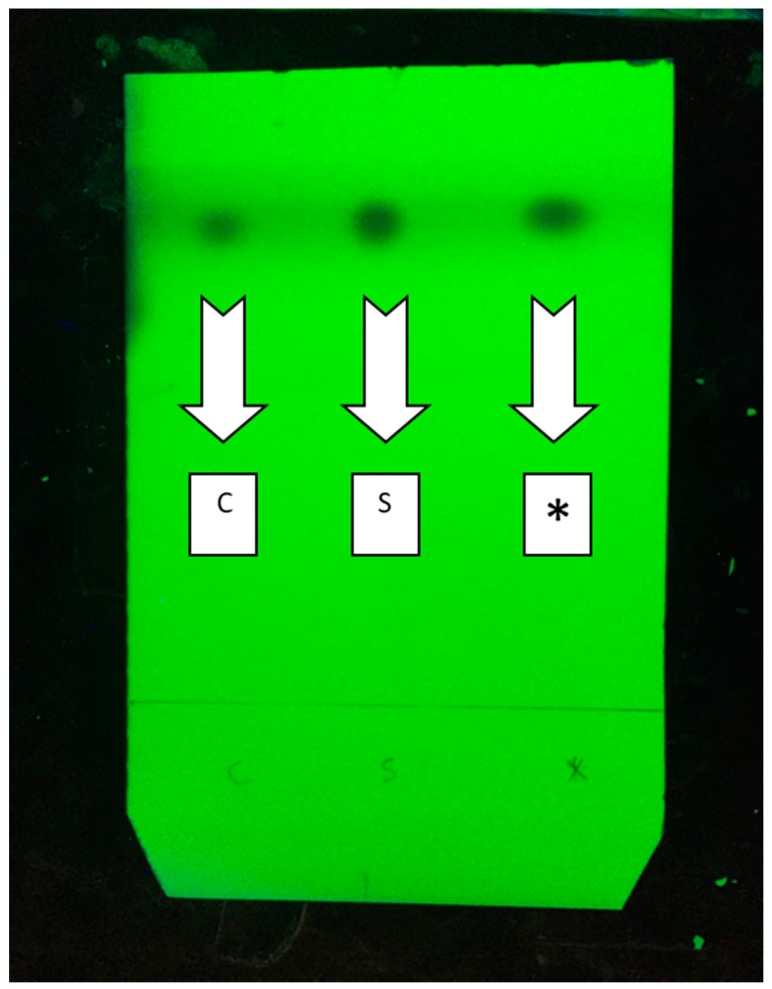
A mix probe between synthesized (*Z*)-2,3-diphenylacrylonitrile and C1 fraction in TLC. C: Possible main active ingredient of C1 fraction. S, Synthesized (*Z*)-2,3-diphenylacrylonitrile; *, both the possible main active ingredient of C1 fraction and synthesized (*Z*)-2,3-diphenylacrylonitrile.

As shown in Figure 8, we made a mix probe between synthesized (*Z*)-2,3-diphenylacrylonitrile and C1 fraction and confirmed that both parts of mix probe run as one spot using Thin Layer Chromatography.

## 3. Discussion

Sea cucumbers are a marine source of food in many Asian countries. They have been considered as a source of various active compounds with different biological activities such as anti-tumor, anti-cancer, anti-antigenic, and anti-inflammatory effects. Bioactive molecules, which are responsible for these therapeutic effects, belong to various classes of compounds such as triterpene glycosides, sulfated polysaccharides, sterols, cerebrosides and peptides [8]. In this study, the selective toxicity of Persian Gulf sea cucumber’s (*H. parva*) methanolic sub-fractions on liver mitochondria was evaluated. Mitochondria have an essential role in apoptosis pathways. There are many significant differences between cancerous mitochondria and normal mitochondria in function and structure. It is known that ROS can cause irreversible damage to the cellular constituents. On the other hand, apoptosis and proliferation are mediated by increased levels of ROS [4]. Mitochondrial swelling is the well-known indicator of mitochondrial MPT pore opening, the starting event in mitochondria mediated apoptosis. Thus, determination of mitochondrial ROS levels and mitochondrial swelling were used for screening *H. parva* fractions’ effects. Our results demonstrated that fraction C of methanolic extract of *H. parva* induced significant mitochondrial swelling in the mitochondria of cancerous cells and increase ROS formation in a time and concentration dependent manner in mitochondria obtained from HCC hepatocytes compared to fractions B and A. According to these results, fraction C was re-chromatographed and divided into two sub fractions (C1 and C2).

Fraction C1 significantly increased ROS production in a time and concentration dependent manner in mitochondria obtained from HCC hepatocytes compared to the normal group. The mentioned sub-fraction has also increased the mitochondria swelling of the HCC group in the concentrations of 500 and 1000 μg/mL, while the C2 had no significant effect on swelling and ROS formation in mitochondria of the HCC group. The concentrations of 500 and 1000 μg/mL of fraction C1 significantly decreased levels of ΔΨm (mitochondrial membrane potential collapse) in mitochondria obtained from HCC hepatocytes but not normal group. MMP is known as an important factor in mitochondrial activity and apoptosis beginning. Moreover, fraction C1 at 500 μg/mL induced significant release of cytochrome c from the HCC mitochondria. Pre-treatment with the MPT pore-sealing agent (C_S_A) and ROS scavenger (BHT) completely blocked the release of cytochrome c from mitochondria induced by fraction C1. These obtained data support the hypothesis that apoptosis induction by fraction C1 of *H. parva* is due to oxidative stress and depends on the opening of the MPT pore.

The attempt to identify the structure of active molecule in fraction C1 revealed the presence of (*Z*)-2,3-diphenylacrylonitrile, which is also known as α-cyanostilbene in active fraction. Jayatilake et al. reported a stilbene derivative from the Antarotic red sponge *Kirkpatrickia* variolosa [9]. Stilbene derivatives are one of the known bioactive compounds in natural products. This group of compounds has wide spectrum of biological activities such as antioxidant, antibacterial, antifungal and anti-cancer. In 2015, Cai et al. isolated a halogen-containing stilbene derivative in leaves of *Cajanuscajan* (L.) Mill sp. They reported this compound has an anti-proliferative effect against cancer cell lines [10]. One of the hydroxylated stilbene derivatives which is known as Resveratrol and isolated from medicinal plants, was reported as apoptosis-inducing agent, aryl hydrocarbon receptor (AhR) modulator, and human cytochrome P450 (CYP) inhibitor [11,12,13,14]. Tyrphostins (tyrosine phosporylation inhibitors), hydroxylated styrenes was reported as a potential tyrosine kinase inhibitor [11]. In this study, a molecule, (*Z*)-2,3-diphenylacrylonitrile, was identified by mass analysis. Our results showed that this molecule significantly induced ROS formation, collapse of MMP, swelling in mitochondria and finally cytochrome c release only on HCC liver mitochondria. In addition, the results showed that (*Z*)-2,3-diphenylacrylonitrile is responsible for these effects.

## 4. Materials and Methods

### 4.1. Sampling and Extraction

Samples of *H. parva* were collected from the Bandar Abbas on the Persian Gulf coast of Iran. The Samples were cut into pieces of 1–2 cm and extracted by methanol at room temperature for 72 h. The obtained extract was filtered, concentrated under reduced pressure below 45 °C, freeze-dried and kept at −20 °C. Briefly, the recovered body wall was cut into small pieces and the samples were homogenized with a blender, and then suspended. This was followed by successive extractions with methanol (50%) by percolation (72 h for each solvent) at room temperature. After filtration and centrifugation (30,000× *g* at 4 °C for 15 min), the extracts were evaporated under a vacuum at 45 °C with a rotary evaporator. The powdered extracts of each sample were obtained with a freeze dryer and stored at −20 °C [4,7].

### 4.2. Fractionation

Thin layer chromatography (TLC) was performed on pre coated silica gel 60 F254 (20 cm × 20 cm; 0.1 mm) plates from Merck (Darmstadt, Germany). The methanolic extract was separated on TLC plate using MeOH:CHCl_3_ (7:3) as eluent into three UV-distinct bands. The active fraction was re-chromatographed on TLC plate with MeOH:CHCl_3_ (7:3) to obtain sub-fractions.

### 4.3. Molecular Identification

Mass spectrometry (Agilent, Santa Clara, CA, USA) is an analytical method in searching for quantitative and qualitative measures in molecular weight and structure for both organic and inorganic compounds. The first component of the stated technique is sample inlet, which brings the sample to the lower pressure of the mass spectra. The sample inlet leads to the ion source where the sample molecules are transformed into gas phase ions which are accelerated by an electromagnetic field, then these are separated based on their mass-to-charge (*m*/*z*) ratio. The ions are then counted by the detector, and the signal is processed by data system.

Electron ionization (EI) mass spectra is an ionization method in which energetic electrons interact with solid or gas phase atoms and molecules to produce ions [9]. The structure of the component in active fraction was determined by EI mass spectra (70 eV) using an Agilent Triple Quad 7000 series (Agilent, Santa Clara, CA, USA) with direct inlet probe. The ion source temperature was maintained at 230 °C.

### 4.4. General Procedure for Preparation of (Z)-2,3-Diphenylacrylonitrile

Benzyl cyanide (2.0 mmol) was added to benzaldehydes (2.5 mmol) in ethanol (15–20 mL), after which the mixture was stirred at room temperature for 10–15 min. To this solution, sodium ethoxide (0.7 g, 10 mmol) in the same solvent (10 mL) was added drop wise with constant stirring, and the reaction mixture was vigorously stirred for 20–72 h to complete the reaction, which was monitored by TLC. After removal of the solid materials and solvent, crude products were purified by silica gel column chromatography (eluent: mixtures of dichloromethane and n-hexane from 1:1 to 7:3 gradient) [11].

### 4.5. Animals

Male wistar rats were used in all experiments. The rats have free access to standard chow diet and water ad libitum. They were kept under 20–25 °C room temperature and 50% humidity and exposed to 12-h light/dark cycles. All experiments were performed according to the ethical standards and protocols approved by the Committee of Animal Experimentation of Shahid Beheshti University of Medical Sciences Tehran, Iran.

### 4.6. Experimental Design

The experiment conducted on two groups of five rats. One group served as the normal control without any treatment and in the other group, HCC, was induced by a single intraperitoneal (i.p.) injection of diethylnitrosamine (DEN) at a dose of 200 mg/kg. After two weeks, cancer was promoted with dietary intake of 2-acetylaminofluorene (2-AAF) (0.02% *w*/*w*) for two weeks [15].

### 4.7. Isolation the Mitochondria from Hepatocytes

Liver cells of rats were obtained by two-step collagenase liver perfusion techniques [4]. To obtain mitochondria, the liver cells were treated according to reported method by seydi et al. [4]. The final mitochondrial pellets were suspended in Tris buffer (0.05 M Tris-HCl, 0.25 M sucrose, 20 mM KCl, 2.0 mM MgCl_2_, and 1.0 mM Na_2_HPO_4_; pH = 7.4) at 4 °C. To assess ROS production mitochondrial pellets were suspended in respiration buffer (0.32 mM of sucrose, 10 mM of Tris, 20 mM of Mops, 50 μM of EGTA, 0.5 mM of MgCl_2_, 0.1 mM of KH_2_PO_4_, and 5 mM of sodium succinate) [16,17]. For determination of MMP the mitochondrial pellets were suspended in MMP assay buffer (220 mM of sucrose, 68 mM of d-mannitol, 10 mM of KCl, 5 mM of KH_2_PO_4_, 2 mM of MgCl_2_, 50 μM of EGTA, 5 mM of sodium succinate, 10 mM of HEPES, and 2 μM of rotenone) and for determination of mitochondrial swelling the mitochondrial pellets were suspended in swelling buffer (70 mM of sucrose, 230 mM of mannitol, 3 mM of HEPES, 2 mM of Tris-phosphate, 5 mM of succinate, and 1 μM of rotenone) [4,18].

### 4.8. Determination of Protein Concentration

Protein concentrations were determined with the Coomassie blue protein-binding method as explained by Bradford [19]. The isolation of mitochondria was confirmed by the measurement of mitochondrial complex II (succinate dehydrogenase) activity [18,20]. In the current research to dissolve (*Z*)-2,3-diphenylacrylonitrile, we used a vehicle solvent containing 30% methanol, 70% water in a total volume of 200 μL. After addition of vehicle treatment containing (*Z*)-2,3-diphenylacrylonitrile to the 1800 μL mitochondria suspended incubation buffer, no precipitation (or any change in solubility) was observed. The concentrations of (*Z*)-2,3-diphenylacrylonitrile (10, 20, 40 μg/mL) were chosen based on the common mitochondrial protocol which suggested using IC_50_/2, IC_50_ and 2 × IC_50_ for toxicological studies. Our control mitochondria, which is present in all of our experiments, is indeed vehicle treated mitochondria. The isolated mitochondria are usually suspended in a combination of 1800 μL incubation buffer plus 200 μL of vehicle which is supposed to be used for dissolving test substance (final volume of mitochondrial suspension is 2 mL).

### 4.9. Determination of Mitochondrial ROS

For determination of mitochondrial ROS, the isolated mitochondria from both normal and the HCC groups were suspended in respiration buffer. In addition, dichlorodihydrofluorescein-diacetate (DCFH-DA) as fluorescent probe was added (final concentration, 10 μM). The suspension was incubated for 10 min at 37 °C. The fluorescence intensity of dichlorofluorescein (DCF) was measured using the Shimadzu RF-5000 U fluorescence spectrophotometer (Shimadzu Corporation, Tokyo, Japan) at an excitation and emission wavelength of 488 nm and 527 nm, respectively [4,7].

### 4.10. Determination of Mitochondrial Swelling

The mitochondria, which were suspended in mitochondrial swelling buffer, were added to 96-well plates and incubated with 250, 500 and 1000 μg/mL of *H. parva* extract fractions and 10, 20 and 40 μg/mL (*Z*)-2,3-diphenylacrylonitrile. The mitochondrial swelling was monitored using ELISA reader (Tecan, Rainbow Thermo, Grödig, Austria) at wavelength of 540 nm for 1 h. The decrease in absorbance indicated an increase in mitochondrial swelling [4,7].

### 4.11. Determination of Mitochondrial Membrane Potential (MMP)

The mitochondria suspensions were incubated with rohdamine 123 (10 μM) in MMP assay buffer [16,17]. The fluorescence intensity was determined at 5, 15, 30, 45 and 60 min by Shimdazu RF-5000 U fluorescence spectrometer at the excitation and emission wavelength of 490 and 535 nm, respectively [4,21].

### 4.12. Determination of Cytochrome c Release

The release of cytochrome c was determined by Quantikine Rat/Mouse Cytochorme c Immunoassay kit (R&D Systems, Inc., Minneapolis, MN, USA) according to previously reported method [7].

### 4.13. MTT Assay

Cell viability was evaluated using MTT assay. At first, hepatocytes were isolated from the rat model of HCC and then placed in 96-well plates (1 × 10^4^ cells/well). Hepatocytes were treated with 0, 2.5, 5, 10, 20, 40 and 100 μg/mL concentration of (*Z*)-2,3-diphenylacrylonitrile for 24 h (cells were maintained in RPMI 1640, supplemented with 10% FBS and antibiotics (50 U/mL of penicillin and 50 μg/mL streptomycin). After incubation for a specified time at 37 °C in a humidified incubator, 3-(4,5-dimethylthiazol-2-yl)-2,5-diphenyl tetrazolium bromide (MTT) (5 mg/mL in RPMI 1640) was added to each well and incubated for 4 h after which the plate was centrifuged at 1800× *g* for 5 min. at 4 °C. The buffer solution (containing (*Z*)-2,3-diphenylacrylonitrile) was then removed from the wells by aspiration. After careful removal of the medium, 100 μL of buffered DMSO (to dissolve the resulting formazan crystals) was added to each well, and plates were shaken. Then, absorbance was recorded at 570 nm in a microplate reader [17,22,23].

### 4.14. Statistical Analysis 

The determinations were performed in triplicate and the results are presented as mean ± SD. Statistical significance was determined using one-way ANOVA test, followed by the post-hoc Tukey test or two way ANOVA and Bonferroni post hoc test when required. Statistical significance level was set at *p* < 0.05.

## 5. Conclusions

We have reported previously that methanolic extract obtained from *H. parva* and *H. oculata* could selectively induce apoptosis in the cancerous mitochondria [4]. In this study, the methanolic extract of *H. parva* was investigated thoroughly and the active fraction has been identified. In this fraction, the (*Z*)-2,3-diphenylacrylonitrile molecule, which is also known as alpha-cyanostilbene, was identified by mass analysis. The derivatives of α-cyanostilbene in other natural products are reported as anti-cancer agents. Our results show that (*Z*)-2,3-diphenylacrylonitrile can have anti-cancer effects. Further investigations by means of clinical trials are required to test for their clinical appliance.
